# Sorption ability of the soil and its impact on environmental contamination

**DOI:** 10.2478/intox-2014-0025

**Published:** 2015-03-04

**Authors:** Šárka Hřibová, Helena Zlámalová Gargošová, Milada Vávrová

**Affiliations:** Institute of Chemistry and Technology of Environmental Protection, Faculty of Chemistry, Brno University of Technology, Brno, Czech Republic

**Keywords:** soil, FEAs, infiltration, filtrate, ecotoxicity

## Abstract

From the physical point of view, soil is a heterogenic polydisperse system. It often becomes a place of a secondary contamination during extinguishing uncontrolled areal fires in nature. Foam extinguishing agents (FEAs), used at these events, basically contain surface active substances and perfluorinated compounds. These tend to be captured in the soil matrix due to their specific properties. Contaminants could be partly flushed out with rainwater, which causes several times dilution of contamination and lower ecotoxic activity. However in the dry season, foam solution infiltrates into the bed soil without any dilution. This study deals with the direct influence of soil the sorption complex on ecotoxicity of five selected FEAs, *i.e.* Expyrol F 15, Finiflam F 15, Moussol APS F 15, Pyrocool B and Sthamex F 15. The substances tested were prepared in concentration of work solution and then applied on standard soil matrix LUFA 2.3. For experimental purposes, a column infiltration apparatus was designed and compiled. Filtrates were collected and then tested using the plant organisms *Sinapis alba* and *Allium cepa* L. The study compared ecotoxicologic effects of filtrates with an original work solution. Moussol APS F 15 seems to be the least ecotoxic of the FEAs tested. A direct influence of soil sorption complex onto ecotoxicity reduction was also established. This finding demonstrates the sorption ability of soil particles and ion exchange activity of the soil matrix. It is a positive finding for biota of aquatic environment, yet at the expense of those in soil.

## Introduction

Soil is the most complicated environmental compartment. It consists of solid organic and inorganic material, soil water and gas and, of course, biotic components. All three environmental compartments (water, air, soil) are linked (Vavříček & Kučera, 2014). Due to this fact, the majority of contamination comes into the soil bed. A wide range of man-made chemicals is then partly or totally captured there. It depends on the physicochemical properties of soil whether the contaminant will be captured or somehow changed. The sorption, sequestration, biodegradation, volatilization, leaching, desorption and the uptake of plant and soil organisms, which contribute to the further fate of contaminants in the soil environment. All processes mentioned act on the contamination concurrently in dependence on the nature of pollution and the soil matrix (Domene *et al.,* 2010). Contaminants like surfactants affect biological and physical properties of soil (Koehler *et al.,* 2004).

It should be noted that the soil sorption complex affects the contaminant fate in the environment in dependence on its chemical nature. For example, mostly affected are non-polar substances bound on solid soil particles or substances with ion-exchange activity. Non-polar substances are of lipophilic nature and they can be captured in the soil bed as they are passing through it. Ion active substances can exchange ions from bonding groups and thus change their own character. All these processes can influence the bioavailability of the contaminant and also its toxic activity (Ruggiero *et al.,* 2002). In addition, hardly controllable environmental conditions, such as climate, can exert significant effects on the behavior of contamination. An example is the comparison of the dry and the rainy season (Ruggiero *et al.,* 2002).

Environmental contamination can be assessed in several ways. Risk assessment studies involve both biomonitoring and ecotoxicology testing. Basically, they are most frequently used for testing surface and ground waters and also soils. In the case of soil testing, we have two possibilities. The soil matrix can be assessed directly via contact testing using soil organisms or via water leachate testing using water organisms.

Contact test arrangements are simple with clear results. The direct influence of contaminated soil can be thus evaluated (Domene *et al.,* 2010; Lors *et al.,* 2011). In contrast, water leachate testing offers information about contamination dispersion, its solubility and, vice versa, about the ability of the soil to capture individual contaminants (Lors *et al.,* 2011). In the Czech Republic, water leachates have to be prepared according to standard guidelines issued by the Ministry of Environment of the Czech Republic. The manual is harmonized with ISO (2007). During leachate preparation, a ten-time dilution of contamination concentration occurs (ISO, 2007; Methodological Guideline of the Waste Department to Determine the Ecotoxicity of Waste, 2007). This step causes a particular deviation and defacement of the real fate of the contaminant. It is necessary to count with the dilution factor to obtain approximate information about ecotoxic activity. Nevertheless, it could be associated with a high error. This may be the reason why bioassays with water extracts are poorly predictive during assessment of environmental risks of soil contamination (Lors *et al.,* 2011).

This hypothesis naturally leads to the effort of designing an experiment for assessment of the influence of the soil matrix on contamination by the infiltration process.

## Materials and methods

### Soil matrix

The standard soil matrix LUFA 2.3 (LUFA Speyer Germany) was used. It is naturally occurring soil in a selected area, which is simultaneously used in agriculture. LUFA 2.3 is classified as sandy loam soil type in accordance with the United States Department of Agriculture (USDA).

The supplier warrants that biocidal fertilizers, organic manure, or pesticides have not been applied for five years at least. Soil can not be sampled earlier than three months after application of mineral fertilizers. Samples are taken from the depth of 20 cm and sieved through a 2 mm sieve. The supplier provides information about soil composition and properties. Some of them are listed in Table 1 (LUFA Speyer, 2013).

**Table 1:** Mean values of LUFA 2.3 analysis with standard deviation referred to dry matter.

Parameter	Unit	Value
Nitrogen	% N	0.08±0.02
pH-value	0.01M CaCl_2_	6.80±0.20
CEC	meq/100g	10.9±1.10
WHC	g/100g	37.3±1.80
Organic carbon	% C	0.94±0.10

CEC – cation exchange capacity; WHC – water holding capacity; meq - miliequivalent

The advantage of this type of soil matrix lies in its similarity with the natural soil ecosystem. Artificial soil provided by OECD 207 can not provide this quality.

### Agents tested

In this study, fire extinguishing agents (FEAs) were tested. These substances are complicated mixtures composed mainly of alcohols, surfactants and other additives. Some of them contain perfluorinated compounds, which belong to persistent organic pollutants (POPs) with relatively high environmental toxicity. Surfactants contained in such a mixture can exchange some ionic groups with soil sorption complex. Perfluorinated compounds can be then adsorbed on solid soil particles, clay surfaces and abiotic organic matter such as humic substances. Adsorption on microbial biofilms is also possible (Lors *et al.,* 2011).

We chose five FEAs for our experiments, i.e. Expyrol F-15 (EXP), Finiflam F-15 (FIN), Moussol-APS F-15 (MOU), Pyrocool B (PYR) and Sthamex F-15 (STH). These FEAs are used by the Fire Rescue Unit of the South Moravian Region of the Czech Republic.

For experimental purposes, FEAs were diluted with deionized water to concentrations of commonly used work solutions, i.e. 5% concentration for STH and 3% for the other ones. Solutions were used as stock solutions and tested for hazardous property H14 (ecotoxicity).

### Experimental design

**Figure 1:**
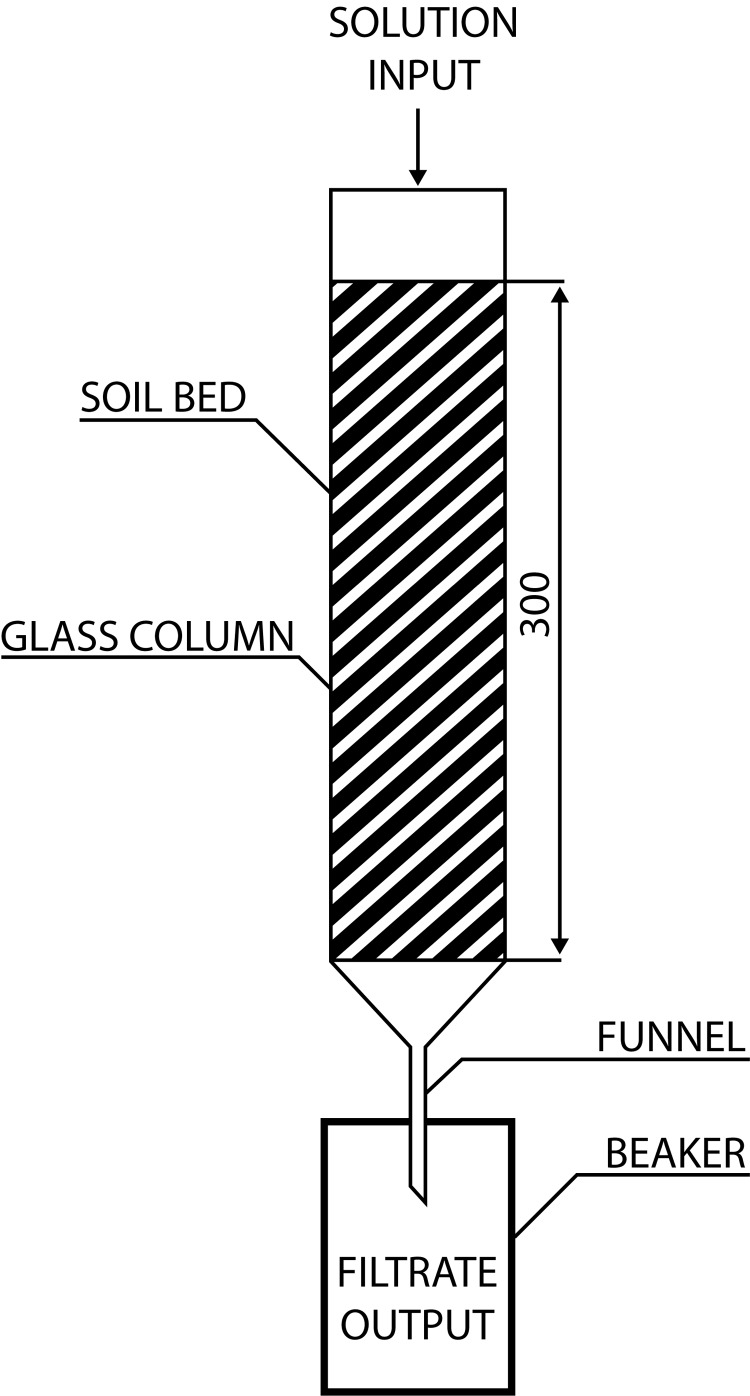
Technical scheme of the designed apparatus. Dimensions are given in units of mm.

At the beginning of our experiment, the column apparatus for infiltration was designed. It was compiled from a glass cylinder with specific proportions (see Figure 1). Standard soil matrix was placed in such a way that it reached average height, which corresponds to topsoil height and also height used for infiltration *in-situ* experiments (Hirzel & Matus, 2013; Johnson, 1963; Mijangos & Garbisu, 2010). Work solutions of the FEAs tested were individually applied in exact volume onto a thus prepared matrix. The out-coming filtrates were collected and then tested via ecotoxicology tests. Work solutions were tested in the same way as their filtrates.

### Column preparation

The bottom of the column was fixedly isolated by sterile gauze. Standard soil matrix was added in order to reach 30 cm height after moderate shaking down. An identical amount (736.5 ± 1.1 g) of soil matrix was used for every infiltration process.

### Filtrate preparation

Filtrates were prepared by using FEAs stock solutions. Every single solution was poured into the prepared column and left to infiltrate spontaneously. Decrease of the soil column occurred at the end of the infiltration.

Properties of work solutions and filtrates are presented in Table 2. Parameters of stock solutions were measured before infiltration, parameters of filtrates after the process. Accurate experimental conditions are listed in Table 3.

**Table 2:** Fundamental properties of stock solutions and filtrates.

Sample type	Stock solution		Filtrate	
Parameter	pH	γ (µS/cm)	pH	γ (µS/cm)
EXP	7.118	1051	6.043	772
FIN	7.430	830	5.442	1049
MOU	6.643	283	5.017	469
PYR	7.375	1492	4.638	1566
STH	7.530	1522	5.849	1258

γ – electrical conductivity

**Table 3:** Experimental conditions for infiltration and experiment outputs.

FEA	Soil weight (g)	IH of soil bed (cm)	FH of soil bed (cm)	Volume of applied solution (mL)	Filtrate volume (mL)
EXP	735.10	30±0.5	24.5±0.5	1 000	850
FIN	737.85	30±0.5	24.0±0.5	1 000	850
MOU	735.34	30±0.5	24.0±0.5	1 000	850
PYR	737.30	30±0.5	24.5±0.5	1 000	850
STH	736.70	30±0.5	24.0±0.5	1 000	850

IH – initial height, FH – final height

### Ecotoxicological assessment

Ecotoxic activity of the prepared samples was evaluated using the plant organism white mustard *S. alba* and the higher plant *A. cepa* L.

### *Sinapis alba* root growth inhibition test

The test was performed in standard arrangement according to the Methodological Guideline No. 11/2007 of the Department of Waste of The Ministry of Environment of the Czech Republic for the determination of waste ecotoxicity. The principle of the test lies in observation of inhibition or stimulation of mustard root growth. Thirty mustard seeds were exposed to several concentration levels of the samples tested and the length of germinated roots was measured after 72 hour exposition.

Seeds were applied at filtrate paper, moistened with relevant solution, in Petri dishes of about ten centimetre diameter. Petri dishes were placed into an incubator under dark condition and constant temperature of 20 °C for 72 hours. At the end of the test, the length of germinated roots was measured. Two replicates were done for every concentration level, including control.

The control test was performed simultaneously with every assay. Standard dilution medium was used in the control. It is a mixture of four essential salts, namely calcium chloride dihydrate in final concentration 11.76 g/L, magnesium sulphate heptahydrate 4.93 g/L, sodium bicarbonate 2.59 g/L and potassium chloride 0.23 g/L (Methodological Guideline of the Waste Department to Determine the Ecotoxicity of Waste, 2007).

### Test inhibition of root elongation of onion sets *Allium cepa* L.

The assay principle is assessment of inhibition, or stimulation, on the basis of comparison of root length in toxicant solution with the length in control test. Six onion sets were exposed to several concentration levels both of FEAs and their filtrates. The length of germinated roots was measured after seven days. To obtain more precise results, the roots were cut and the total biomass amount was determined for every concentration level.

Onion sets were peeled and rehydrated one day to germination before testing. They were put into tubes and filled with the relevant solution. The tubes with onions were maintained in laboratory conditions under ordinary daylight regimen and 20 °C for seven days. During the test, the solutions tested in tubes were continuously refilled. Finally, the length of germinated roots was measured. The amount of the total biomass was then determined for every single solution. Two replicates were done for every concentration level. The control test was performed simultaneously with every assay in two replicates (Fiskesjö, 1985; Olorunfemi & Ogunsanwo, 2011).

Steinberg medium, used in the Lemna sp. growth inhibition test, was chosen as standard dilution medium. It was used for the dilution of both samples and the control solution (ISO, 2005).

### Statistical analysis

The obtained data were subjected to statistical analysis by using the statistical software GraphPad Prism 6.0 in order to compare the toxicity of filtrates and work solutions.

The data were tested with analysis of variance (one-way ANOVA) to control statistically significant deviation of root lengths between all replicates in every concentration level.

## Results

**Figure 2:**
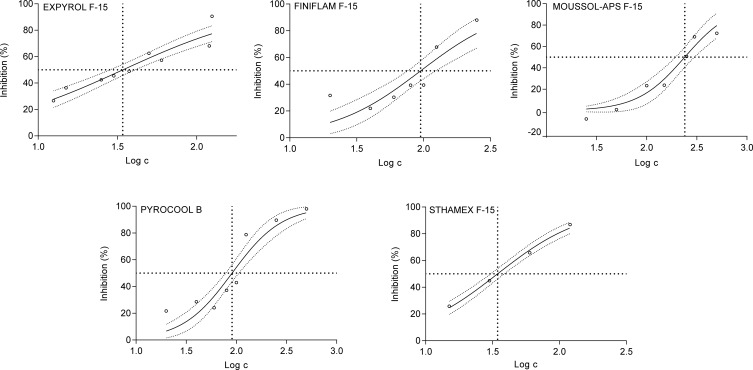
Dose-response curves of individual filtrates, with 95% CI, determined for calculation of 72hIC50 value in the case of *S. alba* testing.

IC50 values, i.e. concentration causing 50% inhibition of root growth, were calculated with 95% interval of confidence using nonlinear regression of dose-response curve as final results of the tests. Figure 2 represents individual dose-response curves for filtrates.

Appropriate results obtained for the organism *S. alba* are listed in Table 4 and for *A. cepa* in Table 5. Listed are only values counted from biomass results in the table. Measuring of root length is not sufficient, due to the high deviation between individual roots on each onion.

**Table 4:** Ecotoxicological effects of the samples tested – *S. alba* root growth inhibition.

FEA	Stock solution			Filtrate		
	LogIC50±SE	72hIC50 (mL/L)	95% CI	LogIC50	72hIC50 (mL/L)	95% CI
EXP	1.496±0.072	31.33	21.40–45.87	1.534±0.036	34.24	28.69–40.85
FIN	1.728±0.079	53.40	36.29–78.56	1.978±0.047	95.03	75.78–119.2
MOU	2.201±0.020	158.8	141.6–178.1	2.380±0.040	239.7	195.8–293.5
PYR	1.364±0.023	23.09	20.63–25.86	1.957±0.031	90.59	78.29–104.8
STH	1.355±0.025	22.63	20.05–25.53	1.538±0.020	34.48	30.74–38.68

CI – confidence interval, SE- standard error

**Table 5:** Ecotoxicological effects of the samples tested – inhibition of *A. cepa* L. root elongation.

FEA	Stock solution			Filtrate		
	LogIC50±SE	168hIC50 (mL/L)	95% CI	LogIC50	168IC50 (mL/L)	95% CI
EXP	1.281±0.102	19.10	9.036–40.39	1.337±0.075	21.74	12.58–37.57
FIN	1.541±0.053	34.75	23.52–51.34	1.545±0.004	35.05	33.71–36.44
MOU	1.884±0.058	76.49	52.77–110.9	2.315±0.605	206.3	OAS
PYR	0.919±0.069	8.293	5.021–13.70	1.186±0.086	15.35	9.244–25.48
STH	1.460±0.065	28.81	15.10–54.98	1.515±0.022	32.76	28.56–37.58

CI – confidence interval, SE- standard error, OAS – outside the acceptable scope

Further, the 72hIC10 (*S. alba* test) values were counted for filtrates. They were compared with 72hIC10 values obtained for commonly prepared leachates, as carried out last year in our previous study (Hřibová *et al.,* 2014). The values were counted by taking into account the dilution factor ten. In no case of leachate testing was 50% inhibition reached. Figure 4 summarizes these results.

## Discussion

The experimental infiltration process had a significant impact on pH reaction and conductivity of solutions (see Table 2). All filtrates had lower pH value than the corresponding stock solutions. On the contrary, conductivity of filtrates compared to stock solutions was elevated in the case of FIN, MOU and PYR. Filtrates of EXP and STH had lower values.

This finding points to the direct influence of the soil matrix on physico-chemical properties of contamination. This is the consequence of combination of many natural processes held in bed soil, climatic conditions and chemical composition of contamination (Lors *et al.,* 2011; Ruggiero *et al.,* 2002).

In the case of assessment of *S. alba* root growth inhibition, Sthamex F-15 proved to be the most toxic agent with 72hIC50 value 22.63 mL/L. Toxicity of the selected FEAs was decreasing in the order STH>PYR>EXP>FIN>MOU.

A somewhat different trend was observed in the case of inhibition of *A. cepa* root elongation testing. Toxic activity of FEAs was decreasing in the order PYR>EXP>STH> FIN>MOU. This time, Pyrocool B was found to be the most ecotoxic agent with 168hIC50 value 8.29 mL/L.

On the other hand, Moussol-APS F-15 proved the least ecotoxic agent in all cases. All agents could fall within the hazard category 3 (concentration ranging 10–100 mL/L; harmful to aquatic life) given by Global Harmonised System (GHS), except Pyrocool B and Moussol-APS F-15. In the case of testing the organism *A. cepa,* Pyrocool B could fall within the category 2 (concentration ranging 1–10 mL/L; toxic for aquatic life). The least toxic agent, Moussol-APS F-15, could not fall within any category in the case of the organism *S. alba* since the IC50 value was higher than 100 mL/L (GHS, 2005).

Indeed, classification of FEAs into GHS categories is complicated, because their current composition is not exactly known. Another option is available. FEAs can be classified by US Fish and Wildlife Service Acute Toxicity Rating Scales, which is used by OECD. According to this classification, Pyrocool B is moderately toxic (IC50 range 1–10 mL/L) for *A. cepa* and Moussol-APS F-15 is practically non-toxic (IC50 range 100–1 000 mL/L) for *S. alba*. The other FEAs fall into the slightly toxic category with IC50 range of 10–100 mL/L (Seow, 2013).

Both classification scales were proposed for acute toxicity testing of water animal organisms such as crustaceans and fish. Thus concerning the effect on plant testing, it can be used only for better imagination about the degree of negative influence of complicated mixtures like FEAs.

**Figure 3:**
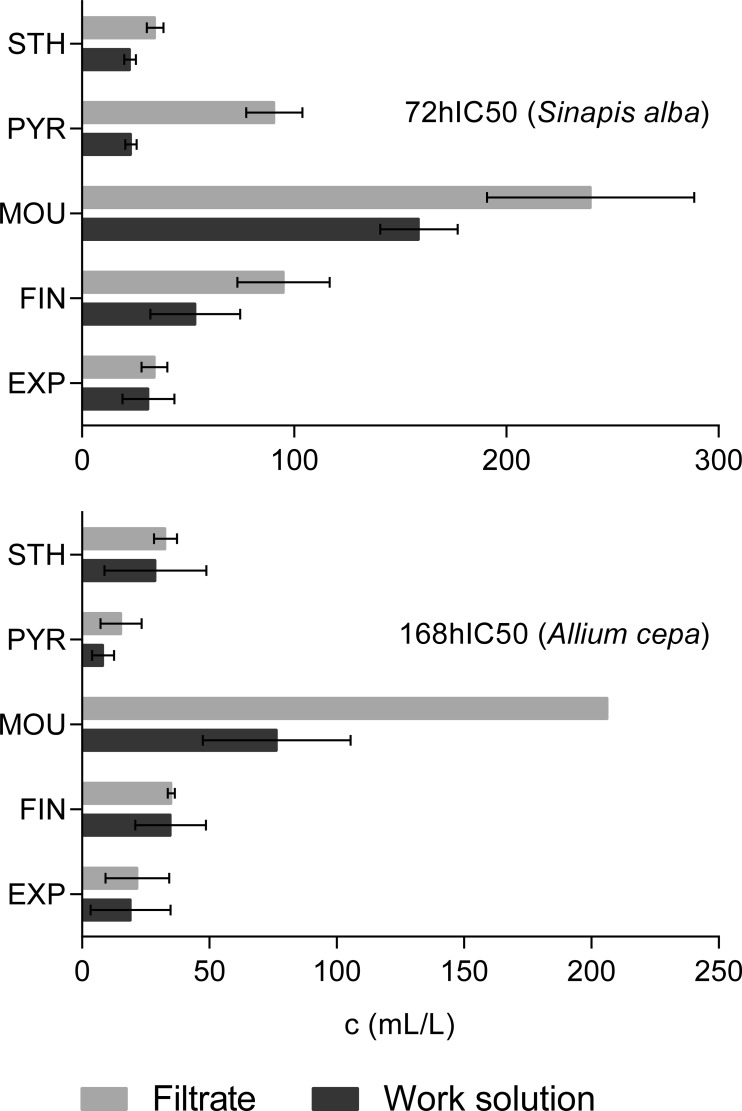
Comparison of ecotoxic activity of work solutions and filtrates of individual FEAs for testing the organisms *S. alba* and *A. cepa* L.

Figure 3 represents the comparison of toxic activity of FEAs work solutions with their filtrates. The figure clearly shows that filtrates affect both testing organisms less than work solutions. This trend is significant in the case of Pyrocool B, Moussol-APS F-15 and Finiflam F-15. Ecotoxicity decrease of the remaining FEAs is weak or almost none.

This finding points to some influence of soil environment on ecotoxicity reduction of contamination. We do not know exactly which physicochemical mechanisms have a major participation in this process. Sorption as well as ion exchange or sequestration could affect the composition of mixtures during the infiltration process (Ruggiero *et al.,* 2002).

Only few studies have so far focused on environmental impacts of FEAs. FEAs are commonly known to be hazardous for organisms of aquatic ecosystems (Adams *et al.,* 2004; Koehler *et al.,* 2004), yet very little is known about their impact on plant species (Adams *et al.,* 2004). Adams *et al.* applied fire-fighting foams used in Australia onto seedlings of higher plant Australian species. They observed no detectable impacts on growth characteristics, flowering and leaf damage (Adams *et al.,* 2004).

Another study was done by Koehler *et al.* This field study provided information about the impact of foam on soil invertebrates, sampled at different times after foam application. Neither did they observe any detectable impact. However the authors admit that further investigation of soil invertebrates at finer taxonomic level may reveal population changes (Koehler *et al.,* 2004).

Ecotoxicity of non-foam fire chemicals was compared with foams in Midwest Science Center in South Dakota. Bioassays were performed with fish, daphnids, amphipods and algae in the study. The results point to a higher toxicity of foams compared with non-foam chemicals used as fire retardants (Hamilton *et al.,* 1996).

Last year, we did an ecotoxic evaluation of FEAs which are the subject of the current work. The results confirmed high risks for aquatic environment and pointed to the influence of soil on ecotoxicity reduction. Conventionally prepared leachates were assessed in that study (Hřibová *et al.,* 2014).

**Figure 4:**
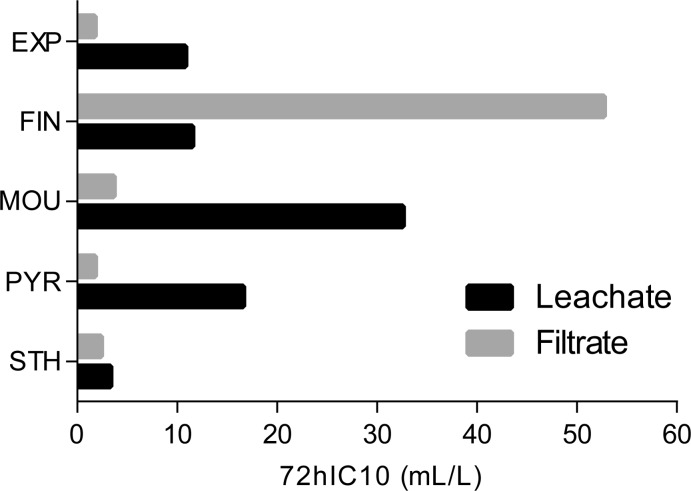
Comparison of 72hIC10 values of convent water leachates and filtrates; test organism *S. alba.*

This year, we compared the results of testing water leachate and filtrates (see Figure 4). A negligible divergence was observed in the case of Sthamex F-15. The remaining FEAs showed considerable variances. It is obvious that two different approaches to soil contamination assessment do not provide the same results. The first and more common possibility is water leachate testing with consideration of the dilution factor. The second one is direct testing of filtrates released from the soil bed after infiltration of a contaminant.

## Conclusion

The present work is focused on the assessment of ecotoxic activity of FEAs. Five foaming agents were chosen, namely Expyrol F-15, Finiflam F-15, Moussol-APS F-15, Pyrocool B and Sthamex F-15. A further aim of the study was rating of the direct influence of the soil matrix on the contamination fate in the environment and subsequently on the reduction of its ecotoxicity.

The results from tests with the plant organisms *S. alba* and *A. cepa* L. provided information about risks for the aquatic environment. Moussol-APS F-15 proved to be the least hazardous agent for both test organisms. Overall, all the FEAs tested are among the slightly toxic and harmful substances according to the GHS and OECD classification systems.

It is important to assess the further fate of fire-fighting foam after its application at the site of fire. Results of two different experimental approaches were compared. Laboratory testing of water leachates prepared according to ISO (ISO, 2007; Methodological Guideline of the Waste Department to Determine the Ecotoxicity of Waste, 2007) was done last year in our previous study. This year, an apparatus for infiltration experiment was designed to assess the direct impact of soil on the contaminant during its passage through the matrix. Filtrates were tested in the same way as leachates. Comparison of the results points to great differences between the approaches.

The message is clear. When assessment of contamination behavior in the environment is needed, it is necessary to consider climatic and further natural conditions. The situation of the rainy season is different from that of the dry one, yet there is no direct division in real ecosystems. Conditions are changing quickly and many factors play a role in contaminant transportation and its transformation. Field studies may provide a better approach in this case.

Finally, in the case of fighting fires, it is important to take into account several factors including economic ones, effectivity and hazards for the environment. Only field studies could promote our understanding of natural processes occurring in a contaminated area and help to decide about the most effective and the least dangerous solutions.
